# Draft Genome Sequence of *Aneurinibacillus* sp. Strain BA2021, Isolated as a Contaminant of a Laboratory-Cultivated Predatory Myxobacterium

**DOI:** 10.1128/MRA.00243-21

**Published:** 2021-04-29

**Authors:** Barbara I. Adaikpoh, Scot E. Dowd, D. Cole Stevens

**Affiliations:** a Department of BioMolecular Sciences, University of Mississippi, Oxford, Mississippi, USA; b MR DNA, Molecular Research LP, Shallowater, Texas, USA; University of California, Riverside

## Abstract

During laboratory cultivation of the myxobacterium Archangium violaceum strain Cb vi76, a reoccurring contaminant was isolated and sequenced. Comparative taxonomic analysis of the draft genome suggested the contaminant to be a novel species, currently designated *Aneurinibacillus* sp. strain BA2021, from the genus *Aneurinibacillus*, members of which are considered promising biocontrol agents.

## ANNOUNCEMENT

The discovery of novel motile spore-forming bacteria of the genus *Aneurinibacillus* offers a promising advancement in the development of sustainable biocontrol agents (1, 2). Significantly, Aneurinibacillus migulanus has been reported to produce the cyclic peptide gramicidin S with activities against a wide range of bacteria and fungi (2, 3). Here, we report a draft genome sequence for *Aneurinibacillus* sp. strain BA2021, which was isolated as a reoccurring contaminant during the laboratory cultivation of a stock of a predatory myxobacterium, Archangium violaceum strain Cb vi76, obtained from the Leibniz Institute DSMZ (German Collection of Microorganisms and Cell Cultures GmbH).

**FIG 1:**
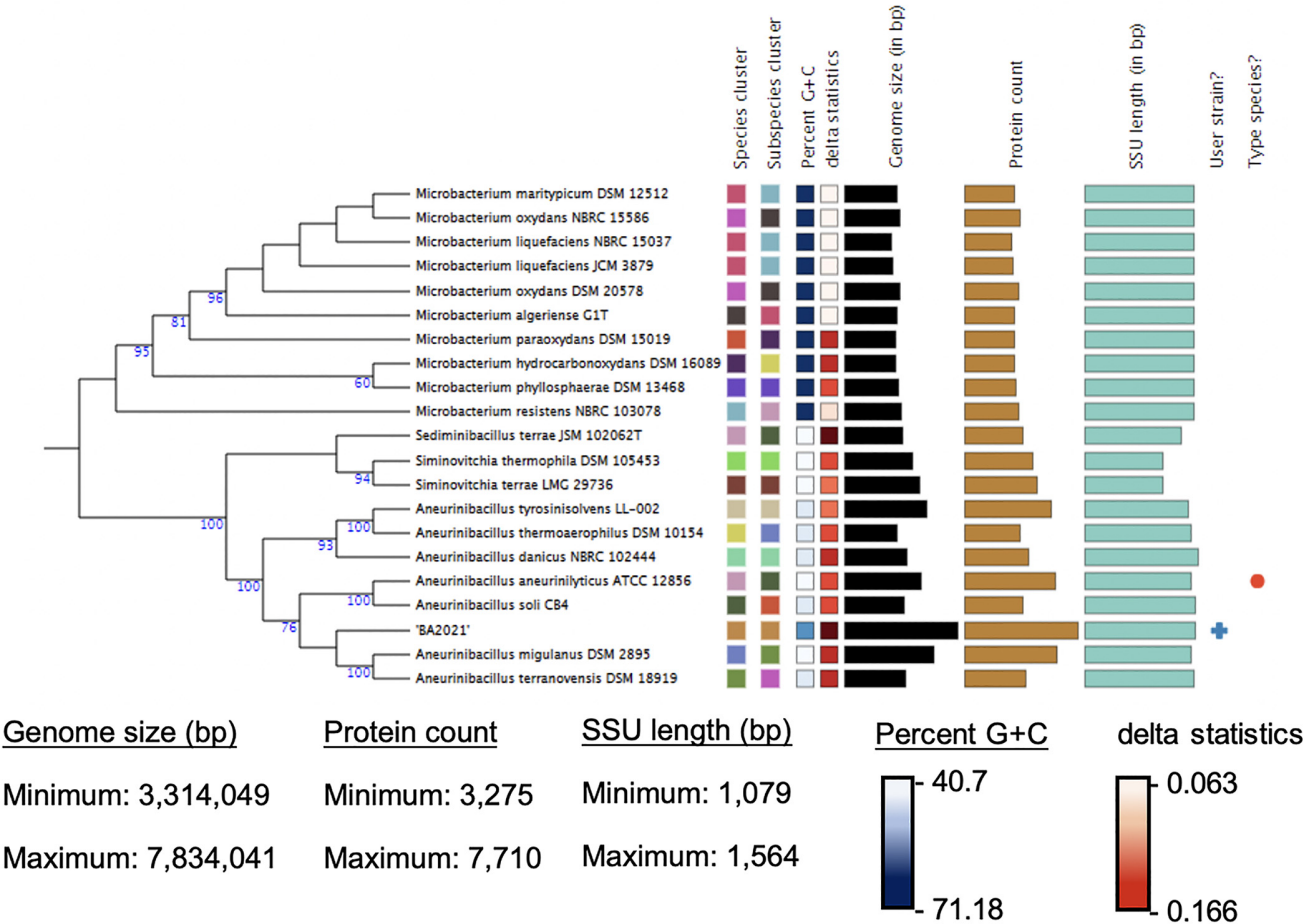
Maximum-likelihood phylogenetic tree suggesting *Aneurinibacillus* sp. strain BA2021 as a member of the genus *Aneurinibacillus*. Clustered species and subspecies are represented by the same colors. The following methods described were obtained from the server: The tree was rendered using the MASH algorithm to compare the draft genome sequence against all type strain genome sequences available in the TYGS database (6). Precise distances were calculated using the genome BLAST distance phylogeny approach (GBDP) with distance formula *d*_5_ to determine closely related type strains. The tree was rendered using FastME version 2.1.6.1 (7) with GBDP distances calculated from 16S rRNA gene sequences, and the numbers on the branches are GBDP pseudobootstrap support values of >60% from 100 replications, with an average branch support of 67.7% (8). SSU, small subunit.

*Aneurinibacillus* sp. strain BA2021 cells were passaged from the originally observed coculture with *A. violaceum* and initially cultivated as a monoculture on VY/2 agar (5 g/liter baker’s yeast, 1.36 g/liter CaCl_2_, 0.5 mg/liter vitamin B_12_, and 15 g/liter agar [pH 7.2]) plates for 8 days at 30°C, transferred to CTTYE broth (10 g/liter Casitone, 5 g/liter yeast extract, 1 mM KH_2_PO_4_, 8 mM MgSO_4_, 10 mM Tris-HCl [pH 7.6]), and incubated as described above. Genomic DNA was isolated from harvested cells using a NucleoBond high-molecular-weight DNA purification kit (Macherey-Nagel), with the initial DNA quantified using the Qubit double-stranded DNA (dsDNA) high-sensitivity (HS) assay kit (Life Technologies) and evaluated using a NanoDrop 2000 spectrophotometer (Thermo Fisher Scientific). Subsequent sample preparation and sequencing were performed at MR DNA (Shallowater, TX). Initial sample sizes of 1 to 15 kb were determined by electrophoresis (E-Gel SizeSelect 2% agarose gel; Invitrogen) and sheared (Covaris g-TUBE) to provide DNA fragments ranging from 6 to 10 kb, which were selected for subsequent sequencing by size using a Sage Science BluePippin automated size selection instrument. A SMRTbell Express template prep kit version 2.0 (Pacific Biosciences) was used to build the library according to the manufacturer’s protocol. Each sample underwent DNA damage and end repair, as well as barcode adapter ligation. After the library construction, the final concentration of the library (8.18 ng/μL) was measured using the Qubit dsDNA HS assay kit (Thermo Fisher Scientific), and the average library size (7,964 bp) was determined using the Agilent 2100 Bioanalyzer. The resulting libraries were sequenced using the 10-h movie time on the PacBio Sequel system. *De novo* assembly of the genome into 3 contigs with protein-coding genes (*N*_50_ contig length, 4,200,366 bp) was accomplished using the SMRT Analysis Hierarchical Genome Assembly Process (HGAP; SMRTLink version 9.0.0), which includes preassembly, assembly, and consensus polishing. Results from the Falcon assembler paired with the Arrow polishing algorithm include a mean seed read length of 10.3 kb (198,513 total reads), 91% realigned bases, and 87.7% mean realigned concordance. A mean coverage of 261× was achieved, and the lengths of the contigs were 4,200,366 bp, 3,559,306 bp, and 74,369 bp. Analysis of the genome completeness, performed using benchmarking universal single-copy orthologs (BUSCO) version 5.1.2 (4) with the “bacillales_odb10” data set with 447 BUSCOs, resulted in 97.3% complete and single-copy, 2% complete and duplicate-copy, 0.4% fragmented-copy, and 0.3% missing BUSCOs. Preliminary annotation, utilizing the Rapid Annotations using Subsystems Technology (RAST) server (5), showed that the draft genome sequence has 58.8% G+C content, 8,498 coding sequences, and a genome size of 7,834,041 bp, containing 216 identified RNAs. Subsequent annotation included with the deposited genome sequence was requested by the NCBI Prokaryotic Genome Annotation Pipeline (PGAP). Taxonomic identification of the genome sequence was performed with the Type (Strain) Genome Server (TYGS) (6) to determine the closest type strain genomes and generate a phylogenetic tree (Fig. 1).

Using antiSMASH version 6.0.0, six unique secondary metabolite biosynthetic pathways were identified, including pathways for two type III polyketides, one terpene, and two β-lactone-containing protease inhibitors (9). However, the biosynthetic pathway for the reported specialized metabolite from this genera, gramicidin S, was not observable in the antiSMASH analysis (10). Ultimately, we envision that the provided genome data will benefit investigations into the utility of members of the genus *Aneurinibacillus* as sustainable biocontrol agents.

### Data availability.

This draft genome sequencing project has been deposited in DDBJ/ENA/GenBank under the accession number JAFJNS000000000. The version described in this paper is the first version, JAFJNS000000000.1. The raw data have been deposited under the BioProject accession number PRJNA698652 and in the Sequence Read Archive (SRA) under the accession number SRR13859926.
